# Supplementary material for: Robo functions as an attractive cue for glial migration through SYG-1/Neph
Source: eLife. 2020 Nov 19;9:e57921. doi: 10.7554/eLife.57921 (PMC7676865; doi:10.7554/eLife.57921)
Supplement: Supplementary file 1. [file elife-57921-supp1.docx]

Supplementary file 1, Strain and plasmid list

| Strain | Genotype | Plasmid | Related Figures |
| --- | --- | --- | --- |
| NYL1269 | *Pf53f4.13::GFP Posm-10::mCherry, Pttx-3::RFP*  *(yadIs48)* | PNYL1124 | Fig. 1 and  WT control for other figures |
| NYL2314 | *sax-3(yad10) X ; Pf53f4.13::GFP Posm-10::mCherry (yadIs48) II* |  | Fig. 1, and all *sax-3* genetic studies |
| NYL2321 | *sax-3(yad147) ; Pf53f4.13::GFP Posm-10::mCherry (yadIs48) II* |  | Fig.1 a, b, c |
| NYL1944 | *sax-3(ky123) X ;Pf53f4.13::GFP Posm-10::mCherry (yadIs48) II* |  | Fig.1 b and c |
| NYL2335 | *slt-1(ok255) X ; Pf53f4.13::GFP Posm-10::mCherry (yadIs48) II* |  | Fig. 1c |
| NYL2332 | *sax-3(yad10) X ; Pf53f4.13::GFP Posm-10::mCherry (yadIs48) II ; Punc-33::sax-3(yadEx1188)* | PNYL 38 | Fig. 1d |
| NYL2333 | *sax-3(yad10) X ; Pf53f4.13::GFP Posm-10::mCherry (yadIs48) II ; Punc-33::sax-3(ΔC)(yadEx1189)* | PNYL 798 | Fig. 1d |
| NYL2334 | *sax-3(yad10) X ; Pf53f4.13::GFP Posm-10::mCherry (yadIs48) II ; Punc-33::sax-3( Δ(C+TM) )(yadEx1190)* | PNYL147 | Fig. 1d |
| NYL2414 | *sax-3(yad10) X ; Pf53f4.13::GFP Posm-10::mCherry (yadIs48) II ; Pf53f4.13::sax-3 (yadEx1243)* | PNYL1296 | Fig. 1d |
| NYL1427 | *Punc-33::sax-3-HA(yadEx692)* | PNYL588 | Fig. 2a and ab |
| NYL1551 | *Punc-33::GFP-FN3 (full)(yadIs59)* | PNYL573 | Fig. 2c |
| NYL1567 | *Punc-33::GFP-FN3 (ΔFN3(2))(yadEx806)* | PNYL666 | Fig. 2d |
| NYL1568 | *Punc-33::GFP-FN3 (ΔFN3(3))(yadEx807)* | PNYL667 | Fig. 2d |
| NYL1428 | *Punc-33::sax-3 (ΔFN3(2)):: HA(yadEx693) line 1* | PNYL1297 | Fig. 2e |
| NYL1470 | *Punc-33::sax-3 (ΔFN3(2))::HA (yadEx736) line 2* | PNYL1297 | Fig. 2e |
| NYL2942 | *Punc-33::sax-3 (ΔFN3(2)):: HA(yadEx693); sax-3(yad10) X ; Pf53f4.13::GFP Posm-10::mCherry (yadIs48) II* | PNYL1298 | Fig. 2f |
| NYL2818 | *Punc-33::SP-FLAG-Ig1-5-FN3 (1-3)(sax-3)::GFP(yadEx1387, sax-3 full length control) ; sax-3(yad10) X ; Pf53f4.13::GFP Posm-10::mCherry (yadIs48) II* | PNYL1299 | Fig. 2f, Fig. 3a |
| NYL2919 | *Punc-33::SP-FLAG-Ig1-5-FN3(1)(sax-3)::GFP(yadEx1389) ; sax-3(yad10) X ; Pf53f4.13::GFP Posm-10::mCherry (yadIs48) II* | PNYL1300 | Fig. 2f |
| NYL2831 | *Punc-33::SP-FLAG-Ig1-5(sax-3)::GFP(yadEx1401) ; sax-3(yad10) X ; Pf53f4.13::GFP Posm-10::mCherry (yadIs48) II* | PNYL1301 | Fig. 2f |
| NYL2832 | *Punc-33::SP-FLAG-FN3(1)(sax-3)::GFP(yadEx1391);sax-3(yad10) X ; Pf53f4.13::GFP Posm-10::mCherry(yadIs48) II* | PNYL1302 | Fig. 2f |
| NYL2692 | *syg-1(ok3640) X; Pf53f4.13::GFP Posm-10::mCherry (yadIs48) II* |  | Fig. 3 c, d, and e |
| NYL2711 | *sax-3(yad10) X syg-1(ok3640) X ; Pf53f4.13::GFP Posm-10::mCherry (yadIs48) II* |  | Fig. 3d and 3e |
| NYL2762 | *Punc-33::sp-FLAG-sax-3-GFP Pmir-228::syg-1-2XHA(yadEx1377)* | PNYL1303  PNYL1304 | Fig. 3b |
| NYL2768 | *Pf53f4.13::syg-1-2XHA(yadEx1381) ; syg-1(ok3640) X; Pf53f4.13::GFP Posm-10::mCherry (yadIs48) II* | PNYL1305 | Fig. 3e |
| NYL2772 | *Pf53f4.13::syg-1(WIRS deletion)-2XHA(yadEx1385) ; Pf53f4.13::GFP Posm-10::mCherry (yadIs48) II ; syg-1(ok3640) X* | PNYL1306 | Fig. 3e |
| NYL2693 | *syg-2(ky671) X; Pf53f4.13::GFP Posm-10::mCherry (yadIs48) II* |  | Fig. 3e |
| NYL2774 | *gex-3(zu196) IV / unc-?(n754) let-?(nT1) ; Pf53f4.13::GFP Posm-10::mCherry (yadIs48) II* |  | Fig. 3f and 3g |
| NYL2829 | *Pf53f4.13::gex-3(yadEx1403);gex-3(zu196) IV / unc-?(n754) let-?(nT1)* | PNYL1307 | Fig. 3g |
| NYL2944 | *Pitr-1(b)::SP-FLAG-Ig1-5-FN3(1)(sax-3)::GFP (yadEx1474); sax-3(yad10) X ; Pf53f4.13::GFP Posm-10::mCherry (yadIs48)* | PNYL1308 | Fig. 3h and 3i |
| NYL2946 | *Pitr-1(b)::SP-FLAG-Ig1-5-FN3(1)(sax-3)::GFP (yadEx1474); sax-3(yad10) X syg-1(ok3640) X; Pf53f4.13::GFP Posm-10::mCherry (yadIs48)* | PNYL1308 | Fig. 3h and 3i |
| NYL3053 | *Psyg-1::GFP Pf53f4.13::H2b::mCherry (yadEx1539)* |  | Fig. 3-Sup. 2a |
| NYL3063 | *Plin-44::sax-3(Ig(1-5)FN3-a)(yadEx1543);sax-3(yad10)X; Pf53f4.13::GFP Posm-10::mCherry (yadIs48)* |  | Fig. 3h and 3i |
| NYL3064 | *Plin-44::sax-3(Ig(1-5)FN3-a)(yadEx1543);sax-3(yad10)X syg-1(ok3640); Pf53f4.13::GFP Posm-10::mCherry (yadIs48)* |  | Fig. 3h and 3i |
| NYL3062 | *Prgef-1::sax-3(Ig(1-5)FN3-a)(yadEx1542);sax-3(yad10)X; Pf53f4.13::GFP Posm-10::mCherry (yadIs48)* |  | Fig. 2-Sup.1d |
| NYL3061 | *Pf16f9.3::sax-3(yadEx1541);sax-3(yad10)X; Pf53f4.13::GFP Posm-10::mCherry (yadIs48)* |  | Fig. 1d |
| NYL3060 | *Parl-13::sax-3(Ig(1-5)FN3-a)(yadEx1540);sax-3(yad10)X; Pf53f4.13::GFP Posm-10::mCherry (yadIs48)* |  | Fig. 2-Sup.1d |
| NYL3051 | *Prab-3::RFP(yadEx1538); Pf53f4.13::GFP Posm-10::mCherry (yadIs48)* |  | Fig. 1-Sup.1d and 1e |
| NYL3052 | *Prab-3::RFP(yadEx1538); Pf53f4.13::GFP Posm-10::mCherry (yadIs48); sax-3(yad10)X* |  | Fig. 1-Sup.1d and 1e |
| NYL3138 | *sax-3(yad175)X; Pf53f4.13::GFP Posm-10::mCherry (yadIs48)* |  | Fig. 2f, Fig. 1-Sup.f, and Fig.2-Sup. 1d |
| NYL3170 | *Pmec-4::GFP(zdId5)I; sax-3(yad175)X* |  | Fig.2-Sup. 1d |
| NYL3171 | *Pf16f9.3::H2b::GFP (yadEx1555)* |  | Fig.1-Sup. 1g |
| NYL3172 | *P f53f4.13::H2b::GFP (yadEx1556)* |  | Fig.1-Sup. 1g |
